# Image-guided lumbar facet joint infiltration in nonradicular low back pain

**DOI:** 10.4103/0971-3026.44522

**Published:** 2009-02

**Authors:** Arti Chaturvedi, Sunil Chaturvedi, Rajiv Sivasankar

**Affiliations:** Department of Radiodiagnosis, Command Hospital, Air Force, Bangalore, India; 1 Department of Anesthesia and Pain Medicine, Command Hospital, Air Force, Bangalore, India

**Keywords:** Facetal arthropathy, facetal infiltrations, low back pain

## Abstract

**Objective:**

To assess the efficacy of facet joint infiltrations for pain relief in 44 selected patients with chronic nonradicular low back pain (LBP).

**Materials and Methods:**

Forty-four patients with chronic LBP of more than 3 months' duration were selected for facet joint infiltration. The majority (n = 24) had facetal pain with no evidence of significant facetal arthropathy on imaging. Fifteen patients had radiological evidence of facetal arthropathy, one had a facet joint synovial cyst, three were post–lumbar surgery patients, and two patients had spondylolysis. Facet joint injections were carried out under fluoroscopic guidance in 39 patients and under CT guidance in 5 cases. Pain relief was assessed using the visual analog scale at 1 h post-procedure and, thereafter, at 1, 4, 12, and 24 weeks.

**Results:**

A total of 141 facet joints were infiltrated in 44 patients over a 2-year period. There was significant pain relief in 81.8% patients 1 h after the procedure, in 86.3% after 1 week, in 93.3% after 4 weeks, in 85.7% after 12 weeks, and in 62.5% after 24 weeks. No major complications were encountered.

**Conclusions:**

Facet nerve block was found to be a simple, minimally invasive, and safe procedure. With meticulous patient selection, we achieved long-term success rates of over 60%. We conclude that this method represents an important alternative treatment for nonradicular back pain.

Chronic low back pain (LBP) has assumed endemic proportions, with an annual prevalence of 5–20% in the industrialized world.[1] It is not always possible to pinpoint the exact structure or pathology responsible for LBP. In 2001, a large study on patients with chronic LBP showed that in as many as 15–45% of patients the pain was due to pathology of the facet joints and only in 13–20% was the pain due to herniated discs.[2,3] With the realization by surgeons that not all backache is a ‘disc’ and not all patients with LBP will respond to surgery, there is increasing awareness of the role of percutaneous injection techniques in the nonoperative management of chronic LBP. The use of image-guidance with fluoroscopy or CT scan has increased the precision and safety of these procedures.[4] However, despite the increasing popularity of these procedures, there are few studies exploring their therapeutic efficacy. We report our experience of facet joint infiltration in 44 selected cases of chronic nonradicular LBP. To the best of our knowledge, this is the first sizeable series documenting the therapeutic use of facet joint injections in the Indian context.

## Materials and Methods

### Patient selection

All patients were initially assessed by a neurosurgeon before referral to the anesthesiologist-run pain clinic. A total of 44 patients were included in the study on the basis of the following criteria:

- Chronic LBP of more than 3 months' duration, not responding to conventional drugs, exercise, and physiotherapy
- Symptoms suggestive of facetal pain; i.e., LBP with or without radiation to the buttocks, thigh, or groin; pain increasing on hyperextension; and pain when initiating movement
- Focal tenderness over the facet joint elicited by digital pressure
- Post–lumbar disc surgery patients with persistent pain and no MRI evidence of arachnoiditis or recurrent disc disease

All the patients had a preprocedure MRI for categorical exclusion of a discogenic cause for the LBP. Evidence of facetal arthropathy on MRI was noted but was not considered an inclusion criterion by itself.

The exclusion criteria were:

- A neurological deficit in the lower limb or a positive sciatic nerve stretch sign (i.e., radicular pain radiating below the knee, elicited by a passive straight leg raise of 60–90°)
- Evidence of nerve root compression at the expected level on MRI
- Clinical or imaging evidence of infection or neoplastic disease
- Possible pregnancy, bleeding diathesis, or anticoagulant therapy
- History of sensitivity to local anesthetics

**Figure 1:**
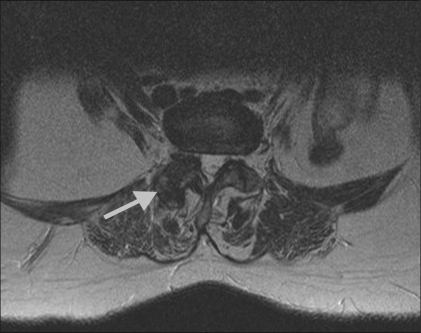
Axial T2W MRI image at L2–3 shows advanced degenerative changes in the right facet joint (arrow).

**Figure 2:**
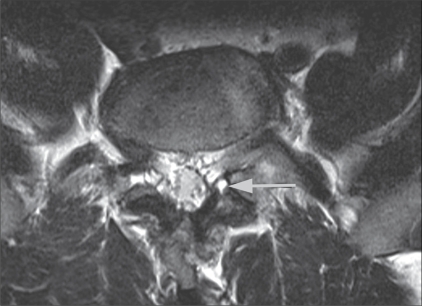
Axial T2W MRI image at L3–4 shows a left facet synovial cyst (arrow).

There were 23 men and 21 women included in the study; the ages ranged from 20–74 years. The duration of symptoms varied from 3 months to 2.5 years (mean duration: 11 months). In our patient population, the largest subgroup (23/44) had clinical signs and symptoms of facetal pain but no evidence of significant facetal arthropathy on imaging (radiography / CT / MRI). Fifteen patients had clinical as well as radiological evidence of facetal arthropathy [Figure 1], one patient had a facet joint synovial cyst [Figure 2], three were post–lumbar surgery patients, and two patients had spondylolysis at the L_4–5_ and L_3–4_ levels, respectively.

### Procedure

The procedure was explained to the patient in detail and written consent was obtained. Injections were performed under fluoroscopic guidance in most (39/44) patients. CT guidance was used in five cases. The levels and side(s) to be injected were selected by the treating pain physician on the basis of the tenderness elicited over the joint, correlated with imaging findings, if any.

**Figure 3:**
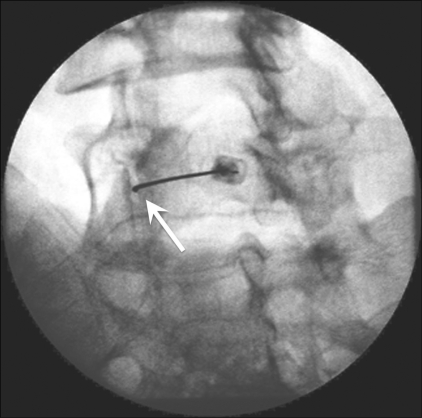
Fluoroscopy-guided lumbar facet joint injection at L4–5. The oblique spot image shows the intra-articular position of the needle (arrow).

*Fluoroscopic procedure:* The patient was placed in the prone position with a pillow under the abdomen to correct the lumbar lordosis. The joint to be injected was located and marked. The x-ray tube was then slowly rotated till the joint appeared in profile as two parallel lines. After cleaning and draping, and administration of local anesthesia, a 22-G spinal needle was inserted in line with the x-ray beam till it contacted bone at the lip of the facet joint. With fine movements the needle tip could be made to enter the joint with a distinct ‘give.’ In the early cases, we confirmed the intra-articular position of the needle by injecting 0.5 ml iohexol (Omnipaque^®^; Amersham Health, New Jersey, USA) under fluoroscopy. Later, with increasing experience, we found that confirmation of correct needle placement could be made by feel and by viewing the joint in the lateral oblique projection [Figure 3]. Once the needle was in place, 0.5 ml of 0.25% bupivacaine (a long-acting local anesthetic) and 0.5 ml (20 mg) of methylprednisolone acetate were injected into the joint. The patient was observed for 1 h after the procedure to document pain relief and to monitor for allergic reactions.

**Figure 4:**
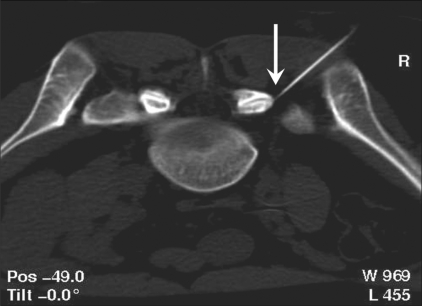
CT-guided facet injection. Axial CT scan in the prone position shows the needle positioned at the lip of the right L5–S1 facet joint.

*CT-guided procedure:* The patient was placed in the prone position and 5-mm axial sections were obtained at the level of interest to determine the entry site and the angle of approach. The entry site was marked on the skin and a 22-G needle was advanced into the joint [Figure 4]. The drug injection protocol was identical to the one used with fluoroscopic guidance.

### Assessment of pain relief

**Figure 5:**
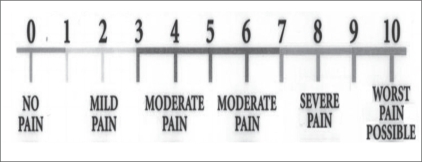
The visual analog scale for pain assessment (Source: Nature Clinical Practice Rheumatology 2007; 3: 610-618)12

Pain relief was assessed using a visual analog scale (VAS), with a score of 0 denoting ‘no pain’ and a score of 10 the ‘worst pain possible’ [Figure 5]. The VAS score was assessed before the procedure, 1 h after the procedure and, thereafter, at 1, 4, 12, and 24 weeks. A reduction in the VAS score of 50% or more from the pretreatment score was considered as significant pain relief and the patient was labeled a ‘responder.’

## Results

A total of 141 facet joints (75 bilateral and 66 unilateral) were infiltrated in 44 patients, over a period of 2 years. The maximum number of infiltrations were at L_4–5_ (31.9%) followed by L_3–4_ (26.5%) [Table 1]. Only a small proportion of the patients (6/44) had an injection at a single level; most required injections at multiple levels: 14 at two levels, 16 at three levels, and 8 at four or more levels.

**Table 1:** Levels of facet joint infiltrations

Level	Bilateral	Unilateral	Total	Percentage
L5–S1	14	16	30	21.3
L4–5	25	20	45	31.9
L3–4	21	16	37	26.5
L2–3	12	11	23	16.3
L1–2	02	02	04	2.7
D12–L1	01	01	02	1.3
Total	75	66	141	100

### Pain relief

The number of patients with significant postprocedure pain relief on day 1 and at 1, 4, 12, and 24 weeks is detailed in Table 2. Immediately postprocedure, 81.8% reported significant pain relief. This number increased slightly at 1 week and reached a peak at 4 weeks, by which time as many as 93.3% patients had responded. However, the number of patients with pain relief declined to 62.5% at 24 weeks.

At 4 weeks, the three patients who did not respond were re-injected at the affected levels. Of these, one responded to the reinjection; the other two remained non-responders throughout the period of follow-up. Four of the patients who had responded well at 4 and 12 weeks required repeat injections between 16 and 20 weeks to maintain the pain relief. Another four patients who had good relief till 12 weeks received repeat injections at 24 weeks as the effect waned.

A further analysis of the pain relief in the different subgroups of patients is shown in Table 3. There was no difference in the number of responders in the group of patients with imaging evidence of facetal arthropathy as compared to those with no imaging findings. The single patient with a synovial facet cyst and both the patients with spondylolysis showed good pain relief in the immediate postprocedure period and remained pain-free for the entire duration of follow-up. The three patients with failed back surgery syndrome (FBSS) showed a poor response, with only one patient responding in the short term; none had any significant pain relief after 4 weeks, despite re-injections.

**Table 2:** Assessment of pain relief after facet injections

Duration following injection	No. of patient ‘responders‘	Percentage	Remarks
1 hour	36 / 44	81.8	
1 week	38 / 44	86.36	
4 weeks	41 / 44	93.3	3 Nonresponders reinjected
12 weeks	36 / 42	85.7	2 Patients lost to follow-up
24 weeks	25 / 40	62.5	4 Patients lost to follow-up
			4 Responders reinjected 16–20 weeks
			4 Responders reinjected at 24 weeks

**Table 3:** Etiology-wise assessment of pain relief after facetal injection

Etiological group	No of patients	No. of patients with significant pain relief				
		
		1 h	1 Week	4 Week	12 Week	24 Week
Clinical facetal pain with no abnormal facet morphology on imaging	23	19	20	22	19/22	13/20
					1 lost to follow-up	3 lost to follow-up
Radiological facetal arthropathy + facetal cyst	16	14	15	16	14/15	10/15
					1 lost to follow-up	1 lost to follow-up
Spondylolysis	02	02	02	02	02	02
FBSS	03	01	01	01	01	0
Total	44	36	38	41	36	25

### Complications

We saw no major complications. Five patients had minor undesired effects in the form of soreness or local skin bruising; these symptoms lasted 2–3 days and subsided without treatment.

## Discussion

Facet joints are true synovial joints which are innervated by the medial branches of the dorsal rami. The presence of nociceptive nerve fibers in the synovium and fibrous capsule of the facet joints suggests that these joints may be a cause for LBP when they are stressed due to segmental instability, inflammatory synovitis, degenerative arthritis, or a combination of all of these.[5,6] Based on studies using controlled diagnostic blocks, it has now been conclusively proved that facet joints are a source of pain in as much as 15–45% of patients with LBP.[2,7]

Unfortunately, there are no clinical or imaging findings to definitively diagnose facetal pain and very often the term ‘facet syndrome’ is used as a ‘dustbin diagnosis’ when nothing else fits. However, there are some features that are characteristic of facetal arthropathy. These include diffuse referred pain over the buttock and posterolateral thigh, exacerbation of pain with hyperextension or lateral bending, tenderness localized over one or more facet joints on deep pressure, and absence of root pain or neurological deficits.[4]

Imaging is not reliable for the diagnosis of facetal osteoarthritis since the changes seen on x-ray, CT, and MRI are equally common in patients with and without LBP, and most studies have failed to show a correlation between radiologic imaging findings and facet joint pain.[8–10]

**Figure 6:**
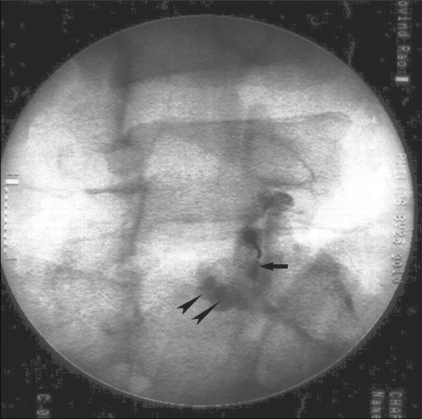
Fluoroscopy-guided lumbar facet joint injection showing intra-articular position of the needle tip and contrast filling the inferior recess (arrow). There is spread of contrast in the perifacetal region (arrowheads) due to rupture of the capsule.

Facet joint injection with local anesthetic and steroid is the simplest and most common procedure for facet joint–mediated pain. These infiltrations are diagnostic as well as therapeutic and the choice of guidance—whether CT or fluoroscopic—is largely a matter of personal preference and experience, as both are equally effective.[4,9,10] The immediate pain relief after the injection is attributed to the effect of the long-acting local anesthetic which interrupts the pain–spasm cycle. The corticosteroid begins to act by 1 week and by about 3 weeks the peak effect sets in. There may be a nonspecific synovitis present in many of these joints that is relieved by the anti-inflammatory action of corticosteroids. In many cases, rupture of the articular capsule during injection results in the drugs diffusing into the neural foramina too, thus, acting on the adjacent nerves as well [Figure 6]. A simple physical effect, whereby inflammatory exudates or adhesions are cleared from the joint and the nerve root sleeve, may also play a role.[11–13]

Accurate assessment of pain is a prerequisite for the effective management of patients with LBP. Of the numerous tools available to assess pain, we adopted the numerical visual analog scale (VAS) since it is an objective measure and can be used to track serial changes.[13] Many recent studies of facet joint injections have also used the VAS as the scoring system for pain, thus allowing us to compare our findings with other studies. Also in accordance with these studies, we considered a 50% reduction in the VAS score from the preprocedure level as indicating significant pain relief.[12,14]

Literature describing the effectiveness of facet joint infiltrations is as abundant as it is controversial. Early studies of facetal infiltrations showed poor results and led to the conclusion that this is a nonspecific and ineffective method of treatment.[14,15] It is possible that these disappointing results were due to improper patient selection, poor localization of the site for injection, or inadequacies in the volumes and types of drugs used.[4] Other studies in recent years have reported encouraging results with facet joint infiltrations and the results of the pain relief obtained in these studies are shown in Table 4. These results demonstrate significant short-term (1–12 weeks) pain relief in 62–74% of patients. Though the pain response gradually declines over time, even in the medium term (up to 24 weeks) as many as one-third of patients still experience significant pain relief.[10,12,17,18] Our results showed short-term relief in 86–93% of patients and medium-term relief in 62%, which compare favorably with the results of these earlier studies. In our opinion, the high number of responders in our study could be due to meticulous adherence to the patient selection criteria, with elicitation of paraspinal tenderness over the facet joint being the most important inclusion criterion. The presence or absence of facet joint arthropathy on imaging was not related to pain relief in any way, and the main factor associated with a successful outcome of facet joint interventions was clinically elicited paraspinal tenderness.[10,12,14]

**Table 4:** Pain relief following lumbar facet injections in various recent studies

Study	Total no. of patients	Percentage of patients with pain relief following facet injection			
		
		1 week	3–4 weeks	12 weeks	24 weeks
Shih *et al.* (2005)[16]	277	73.6%	72.1%	31.4%	Not assessed
Schulte *et al.* (2006)[17]	39	Not assessed	62%	41%	36%
Gorbach *et al.* (2006)[11]	42	74%		33%
Destouet *et al.* (1982)[9]	54	Not assessed	54%		38%

**Figure 7 (A–C):**
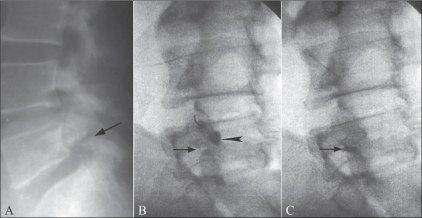
Facet injection in spondylolysis. The lateral radiograph (A) shows a break in the pars interarticularis (arrow). Oblique fluoroscopic spot image (B) shows a fluoroscopy-guided L4–5 facet injection with filling of the inferior recess (arrowhead). Note the spondylolysis (arrow). A more delayed image (C) shows tracking of contrast from the facet joint into the spondylolytic site (arrow). Note the emptying of contrast from the joint space.

Injections for spondylolysis are a modification of facetal injections, providing good response in most patients. Some workers feel that it is the fracture site which is painful and, accordingly, infiltrate the break in the pars interarticularis.[4] Others have demonstrated that injection of the adjacent facet joint also involves the spondylolytic area and the technique can thus be used for therapeutic injections.[10,19] On fluoroscopy, we could demonstrate the passage of contrast from the facet joint into the defect in the pars interarticularis [Figure 7], with good and sustained pain relief. The uniformly poor results with facet joint injections in patients with FBSS is because post–lumbar surgery pain is due to an interplay of numerous causes and facet joint injection addresses only one of them.[14,20] Due to the small number of FBSS patients in our series we could not derive any statistically significant information from our data.

The major complications of facet joint infiltrations are related to improper needle placement, bleeding, or infection. Complications include dural puncture, hematoma formation, spinal cord or neural trauma, spinal anesthesia, septic arthritis / spondylitis, and chemical men-ingitis.[21] Despite this long list and numerous anecdotal reports in literature,[22–24] it is our experience that with meticulous attention to technique and sterility, these major complications can be eliminated.

We acknowledge the following limitations of our study. Firstly, we did not have a control group receiving placebo injections. Secondly, we did not carry out a preliminary diagnostic block for patient selection prior to giving the therapeutic injection. This was because we found it difficult to justify a preliminary diagnostic block followed by a subsequent therapeutic injection; such a protocol would have delayed treatment and exposed the patient repeatedly to the risk of complications and radiation exposure. Therefore we selected our patients on the basis of a good clinical examination.

## Conclusions

Recent evidence-based guidelines, published in 2007, with respect to lumbar facet joint injections have convincingly demonstrated moderate evidence for short- and medium-term (up to 6 months) relief and limited evidence for long-term relief.[11] Facetal injections are not curative; however, by abolishing pain for periods of up to 6 months they can decrease dependence on oral medications and facilitate early return to work. Since their clinical effect is for a limited duration and wanes after 6 months, they need to be repeated to maintain the pain relief. It is also important to adopt stringent criteria for diagnosing facet joint pain in order to avoid unnecessary and unwarranted injections. In conclusion, we have found that in carefully selected cases, lumbar facet block is a relatively simple, safe, and minimally invasive procedure that can be a valuable adjunct in the treatment of LBP.

## References

[1] Cassidy JD, Côté P, Carroll LJ, Kristman V (2005). Incidence and course of low back pain episodes in the general population. Spine.

[2] Manchikanti L, Singh V, Pampati V, Ghafoor AB, Fellows B, Damron KS (2001). Evaluation of the relative contributions of various structures in chronic low back pain. Pain Physician.

[3] Pang WW, Mok MS, Lin ML, Chang DP, Hwang MH (1998). Application of spinal pain mapping in the diagnosis of low back pain: Analysis of 104 cases. Acta Anaesthesiol Sin.

[4] Silbergleit R, Mehta BA, Sanders WP, Talati SJ (2001). Imaging-guided injection techniques with fluoroscopy and CT for spinal pain management. Radiographics.

[5] Boswell MW, Colson JD, Sehgal N, Dunbar EE, Epter R (2007). A systematic review of therapeutic facet joint interventions in chronic spinal pain. Pain Physician.

[6] Cavanaugh JM, Lu Y, Chen C, Kallakuri S (2006). Pain generation in lumbar and cervical facet joints. J Bone Joint Surg Am.

[7] Boswell MV, Shah RV, Everett CR, Sehgal N, Mckenzie-Brown AM, Abdi S (2005). Interventional techniques in the management of chronic spinal pain: Evidence-based practice guidelines. Pain Physician.

[8] Schwarzer AC, Wang S, O'Driscoll D, Harrington T, Bogduk N, Laurent R (1995). The ability of computed tomography to identify a painful zygapophysial joint in patients with chronic low back pain. Spine.

[9] Gilula LA, Lander P (2003). Management of spinal pain with imaging-guided injection. Radiographics.

[10] Destouet JM, Gilula LA, Murphy WA, Monsees B (1982). Lumbar facet joint injection: Indications, technique, clinical correlation, and preliminary results. Radiology.

[11] Boswell MV, Trescot AM, Datta S, Schultz DM, Hansen HC, Abdi S (2007). Interventional techniques: Evidence-based practice guidelines in the management of chronic spinal pain. Pain Physician.

[12] Gorbach C, Schmid MR, Elfering A, Hodler J, Boos N (2006). Therapeutic efficacy of facet joint blocks. AJR Am J Roentgenol.

[13] Mannion AF, Balagué F, Pellisé F, Cedraschi C (2007). Pain measurement in patients with low back pain. Nat Clin Pract Rheumatol.

[14] Cohen SP, Hurley RW, Christo PJ, Winkley J, Mohiuddin MM, Stojanovic MP (2007). Clinical predictors of success and failure for lumbar facet radiofrequency denervation. Clin J Pain.

[15] Lilius G, Harilainen A, Laasonen EM, Myllynen P (1990). Chronic unilateral low-back pain: Predictors of outcome of facet joint injections. Spine.

[16] Nelemans PJ, deBie RA, deVet HC, Sturmans F (2001). Injection therapy for subacute and chronic benign low back pain. Spine.

[17] Shih C, Lin GY, Yueh KC, Lin JJ (2005). Lumbar zygapophyseal joint injections in patients with chronic lower back pain. J Chin Med Assoc.

[18] Schulte TL, Pietila TA, Heidenreich J, Brock M, Stendel R (2006). Injection therapy of lumbar facet syndrome: A prospective study. Acta Neurochir (Wien).

[19] el-Khoury GY, Renfrew DL (1991). Percutaneous procedures for the diagnosis and treatment of lower back pain: Diskography, facet joint injection, and epidural injection. AJR Am J Roentgenol.

[20] Wilkinson HA (1992). Introduction: Etiology, diagnosis, and therapy. In the failed back syndrome.etiology and therapy.

[21] Windsor RE, Pinzon EG, Gore HC (2000). Compli¬cations of common selective spinal injec¬tions: Prevention and management. Am J Orthop.

[22] Magee M, Kannangara S, Dennien B, Lonergan R, Emmett L, Van der Wall H (2000). Paraspinal abscess complicating facet joint injection. Clin Nucl Med.

[23] Weingarten TN, Hooten WM, Huntoon MA (2006). Septic facet joint arthritis after a corticosteroid facet injection. Pain Med.

[24] Berrigan T (1992). Chemical meningism after lumbar facet joint block. Anesthesia.

